# Risks of tinnitus, sensorineural hearing impairment, and sudden deafness in patients with non-migraine headache

**DOI:** 10.1371/journal.pone.0222041

**Published:** 2019-09-06

**Authors:** Yi-Chun Chen, Shiang-Jiun Tsai, Jin-Cherng Chen, Juen-Haur Hwang

**Affiliations:** 1 Department of Nephrology, Dalin Tzu Chi Hospital, Buddhist Tzu Chi Medical Foundation, Dalin, Chiayi, Taiwan; 2 School of Medicine, Tzu Chi University, Hualien, Taiwan; 3 Deparment of Medical Research, Dalin Tzu Chi Hospital, Buddhist Tzu Chi Medical Foundation, Dalin, Chiayi, Taiwan; 4 Department of Neurosurgery, Dalin Tzu Chi Hospital, Buddhist Tzu Chi Medical Foundation, Dalin, Chiayi, Taiwan; 5 Department of Otolaryngology-Head and Neck Surgery, Dalin Tzu Chi Hospital, Buddhist Tzu Chi Medical Foundation, Dalin, Chiayi, Taiwan; 6 Department of Medical Research, China Medical University Hospital, China Medical University, Taichung, Taiwan; Lamar University, UNITED STATES

## Abstract

Tinnitus and hearing impairment are prevalent among headache patients. This study aims to investigate the risk of tinnitus, sensorineural hearing impairment, and sudden deafness in patients with non-migraine headache. Participants included 43 294 patients with non-migraine headache (non-migraine headache cohort) and 173 176 patients with no headache of any type (control cohort) frequency-matched with respect to 10-year age interval and sex from the Longitudinal Health Insurance Database 2005 of the Taiwan National Health Insurance Research Database. The mean age of the non-migraine headache cohort was 28.4 ± 14.9 years, and 58.5% of this cohort was male. The incidence rates of tinnitus, sensorineural hearing impairment, and sudden deafness were compared between cohorts using the Kaplan–Meier method with the log-rank test. A Cox proportional hazard model was used to examine the association of tinnitus, sensorineural hearing impairment, and sudden deafness with non-migraine headache, with adjustment for all covariates. The combined risk of either tinnitus, sensorineural hearing impairment, or sudden deafness was higher in the non-migraine headache cohort than in the control cohort (adjusted odds ratio [aHR], 2.73; 95% confidence interval [95% CI], 2.62–2.84; p < 0.0001). Subgroup analysis showed that patients in the non-migraine headache cohort were at significantly higher risk of developing tinnitus (aHR, 3.05; 95% CI, 2.91–3.19; p < 0.0001), sensorineural hearing impairment (aHR, 1.89; 95% CI, 1.74–2.05; p < 0.0001), and sudden deafness (aHR, 2.14; 95% CI, 1.77–2.59; p < 0.0001) than were controls. In this population-based study, the risks of tinnitus, sensorineural hearing impairment, and sudden deafness were found to be significantly higher in patients with non-migraine headache than in those without headache.

## Introduction

Headache is common in people of all ages. Common chronic headache disorders include migraine, tension-type headache (TTH), and medication overuse headache [1]. Secondary causes of headache, including temporal or giant cell arteritis, subdural hematomas, central nervous system (CNS) tumors, strokes, and central nervous system (CNS) infections, are less prevalent than primary headache in the general population but more prevalent in older adults [2,3]. The prevalence of chronic daily headache in Taiwan (3.2–3.9%) is very similar to that in western countries [1,4]. Among chronic headache patients in Taiwan, the prevalence of TTH is 15.6–25.7% and that of migraine is 8.4–12.7% [4].

Cross-sectional studies have shown that several types of headache are associated with tinnitus and hearing impairment. For example, in 71 patients with tinnitus comorbid with headache, 30 suffered from migraine, 15 from TTH, 5 from cluster headache, and 21 from mixed headache [5]. Among these patients, the tinnitus was bilateral in 66% and involved tones of higher frequencies in 37%, middle frequencies in 11%, lower frequencies in 29% and of multiple frequency ranges in 23% [5]. Another study found that of 193 patients with tinnitus and headache, 44.6% suffered from migraine, 13% from TTH, and 5.7% from both [6]. In 1251 patients with headache and craniofacial pain, tinnitus was found to be associated with cervical and pericranial muscle tenderness but not to any specific type of headache [7]. A population-based cohort study from Taiwan demonstrated that migraine is associated with an increased risk of sudden deafness, most often associated with a specific disease and occurring in one ear [8]. Our previous cohort study found that a history of migraine increases the risks of tinnitus, sensorineural hearing impairment, and/or sudden deafness [9].

The relationship between headache and tinnitus or hearing impairment has been described in serial case and cross-sectional studies [5–7, 10,11]. Cohort studies suggest that migraine increases the risk of tinnitus, sensorineural hearing impairment, and/or sudden deafness [8,9]. However, whether non-migraine headache also increases such risks is unknown. This study aims to address this issue using a nationwide, population-based cohort.

## Materials and methods

### Data source

This retrospective cohort study used claims data from the Longitudinal Health Insurance Database 2005 (LHID2005) of Taiwan, a subset of the National Health Insurance Research Database (NHIRD) [12], derived from Taiwan's National Health Insurance (NHI) program and has been described in detail in our previous studies [13–15]. In brief, Taiwan's NHI program is a single-payer, compulsory program launched in 1995, reaching a coverage rate >99% by the end of 2012. The related database contains comprehensive medical information for all residents of Taiwan and uses ICD-9 codes to classify diseases. The NHIRD was released by the National Health Research Institute before June 28, 2016 after de-identification of all personal information and is freely accessible for academic research.

### Ethical considerations

This study was approved by the institutional review board of the Dalin Tzu Chi Hospital, Buddhist Tzu Chi Medical Foundation, Taiwan (No. B10202022). Because this study uses only de-identified data, the review board waived the requirement for obtaining informed consent from all patients.

### Study population

**Fig 1:**
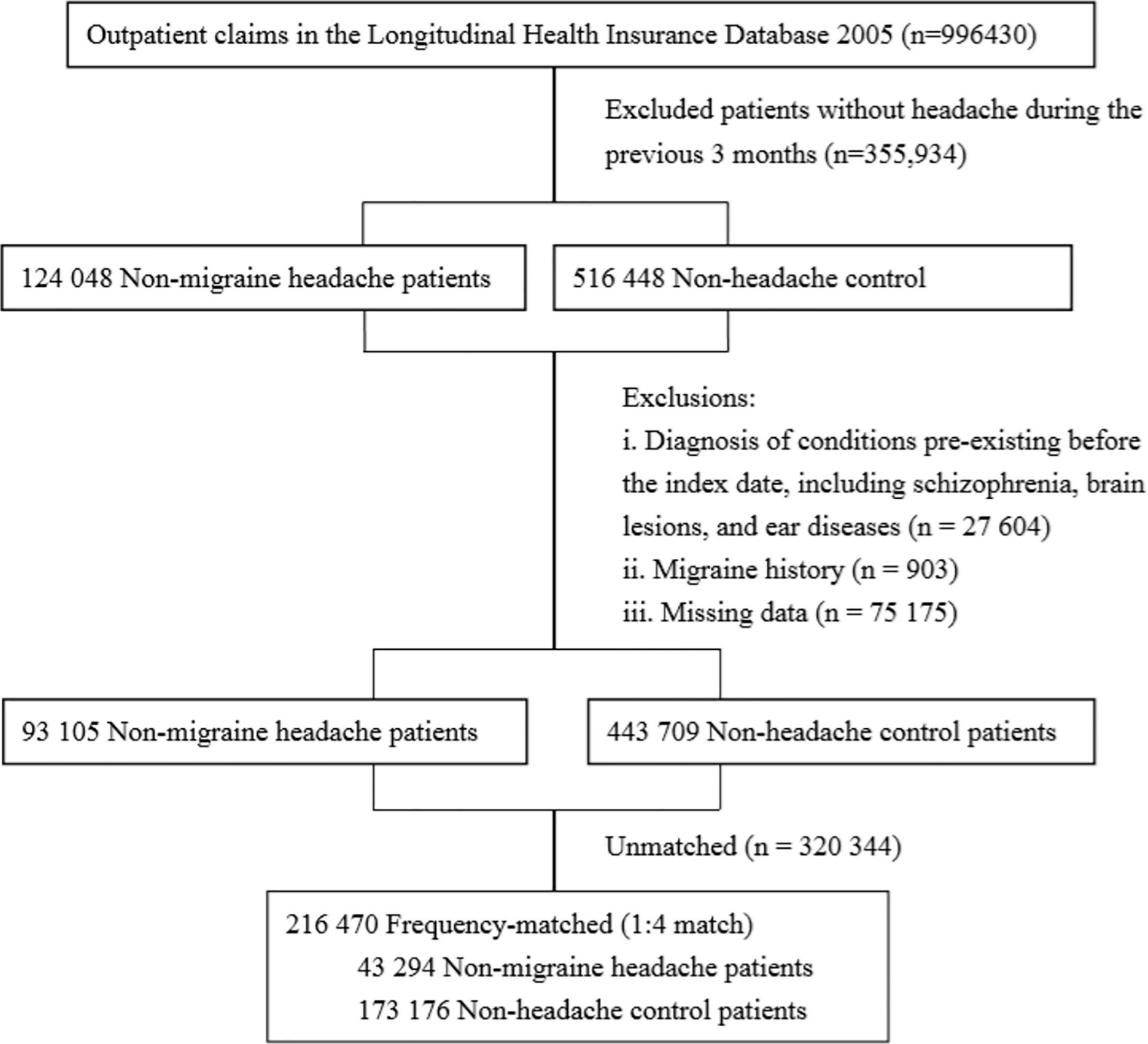
Flow diagram of the enrollment process. We first identified 996,430 patients between 1 January 1996 and 31 December 2012 from the outpatient claim. We excluded patients who had some pre-existing diseases before the index date, had missed data, and could not match well for both cohorts. Finally, a total of 43294 patients with newly diagnosed headache other than migraine were identified as the non-migraine headache cohort. For each patient in the non-migraine headache cohort, 4 control patients were randomly selected, with frequency matching by age and sex.

To investigate whether non-migraine headache might increase the risk of tinnitus, sensorineural hearing impairment, or sudden deafness or not. We first identified 124 048 non-migraine patients with outpatient claims between 1 January 1996 and 31 December 2012 (Fig 1). From this group, we identified patients with non-migraine headache according as determined by the presence of two ICD-9 codes, 784.0 and 307.81, reported within 3 months of each other. The date of the first-ever non-migraine headache diagnosis was considered the index date.

We excluded patients who had several conditions before the index date, including schizophrenia (ICD-9 code 295.90); brain lesion(s), including benign brain tumor (ICD-9 codes 225, 227.3) and malignant brain tumor (ICD-9 codes 191, 192); ischemic cerebrovascular diseases and basilar artery syndrome (ICD-9 codes 433.01, 433.10, 433.11, 433.21, 433.90, 434, 435.0, 435.1, 435.3, 435.8, 435.9, 436, 437.0. 437.8, 437.9, 438.89, 438.9); hemorrhagic stroke (ICD-9 codes 432.9, 432.1, 431, 430, 852.00, 852.40, 852.46); brain concussion or head trauma (ICD-9 codes 800–804, 850–854, 873, 310.2, 907); epilepsy/convulsions (ICD-9 codes 345.9, 345.71, 780.39, 345.40, 345.41, 345.11, 345.10); aneurysm or arteriovascular malformation of brain and neck (ICD-9 codes 747.81, 437.3, 442.81, 442.89); anoxic brain damage (ICD-9 code 348.1); brain abscess/encephalitis (ICD-9 codes 324, 323.9); and ear diseases, including vestibular schwannoma (ICD-9 code 225.1), conductive hearing loss (ICD-9 code 389.0), otosclerosis (ICD-9 code 387.9), tinnitus (ICD-9 codes 388.3, 388.30, 388.31, 388.32), sensorineural hearing impairment (ICD-9 codes 388.12, 388.40, 389.1, 389.2, 389.7, 389.8, 389.9, 389), and sudden deafness (ICD-9 code 388.2). Those with missing or unmatched data and those who had migraine (ICD-9 codes 346.01, 346.10, 346.90) before or after index the date were excluded. Finally, a total of 43 294 patients with newly diagnosed non-migraine headache were enrolled as the non-migraine headache cohort.

Next, we identified 516 448 patients without headache of any type from 1996 to 2012, with exclusion of the above-mentioned pre-existing conditions before the index date for screening as controls. For each patient in the non-migraine headache cohort, 4 control patients were randomly selected, with frequency matching by age and sex. The final matched-control cohort included 173 176 patients. The index date was the date of selection.

### Main outcome measurement

Both cohorts were followed from the index date until the first diagnosis of tinnitus, sensorineural hearing impairment, sudden deafness, death, or the end of 2012, whichever came first. Death was assumed upon withdrawal of a patient from the NHI program [13,15].

### Potential confounders

We identified comorbidities according to ICD-9 codes present before or on the index date, including sleep disorders (ICD-9 codes 780.50, 780.51, 780.52, 780.53, 780.57, 307.40), heart disease (ICD-9 codes 413.9, 414, 410–429, 402), hypertension (ICD-9 codes 401–405), diabetes (ICD-9 code 250), hyperlipidemia (ICD-9 code 272), chronic kidney disease (ICD-9 codes 585, 586), and chronic hepatitis (ICD-9 codes 070, 571.4, 571, 571.2, 571.5, 571.6). Geographic region of residence (northern, central, southern, or eastern Taiwan) and urbanization level (urban, suburban, or rural) were also included to minimize potential confounding due to urban–rural differences in accessibility to medical care in Taiwan.

### Statistical analyses

Pearson’s chi-squared test and Student's t-test were used to compare categorical and continuous variables, respectively, between cohorts. We determined and compared the incidence rates (per 10^6^ person-years) with 95% confidence intervals (CIs) for tinnitus, sensorineural hearing impairment, and sudden deafness using the Kaplan–Meier method between cohorts using the log-rank test. After ensuring the assumptions of proportional hazards, we used the Cox proportional hazard model to examine the association of tinnitus, sensorineural hearing impairment, and sudden deafness, combined and individually, with non-migraine headache, with adjustment for all covariates (age, sex, comorbidities, geographic region, and urbanization level). We analyzed all data using SAS (version 9.4; SAS Institute, Inc., Cary, NC, USA) and SPSS (version 20.0; IBM Corp., New York, NY, USA) and considered a 2-sided p <0.05 statistically significant.

## Results

Table 1 shows compares characteristics between the non-migraine headache and control cohorts by Pearson’s chi-squared test if the variable was categorical, and by Student's t-test if the variable was continuous. The mean age of the non-migraine headache cohort was 28.4 ± 14.9 years, and 58.5% of the cohort was male. The prevalence of comorbidities, geographic region, and urbanization level differed significantly between the non-migraine headache and control cohorts.

**Table 1:** Characteristics in non-migraine headache and control cohorts in Taiwan, 1996–2012 (n = 216,470).

Variable	Non-migraine headache cohort (n = 43,294), N(%)		Control cohort (n = 173,176), N(%)		*p*-value*
Sex					1.00
Men	25348	58.5	101392	58.5	
Women	17946	41.5	71784	41.5	
Age (years, mean±SD)	28.4±14.9		28.4±14.9		1.00
Comorbidities					
Sleep disorders	3558	8.2	54	0.03	<0.001
Heart diseases	4238	9.8	225	0.13	<0.001
Hypertension	3748	8.7	205	0.12	<0.001
Diabetes	1782	4.1	117	0.07	<0.001
Hyperlipidemia	3065	7.1	65	0.04	<0.001
Chronic kidney disease	242	0.6	9	0.01	<0.001
Chronic hepatitis	2305	5.3	77	0.04	<0.001
Geographic region					<0.001
Northern	17435	40.3	96142	55.5	
Central	12413	28.7	35114	20.3	
Eastern	905	2.1	3677	2.1	
Southern	12541	28.9	38243	22.1	
Urbanization level					<0.001
Urban	12153	28.1	60549	35.0	
Suburban	20826	48.1	80638	46.6	
Rural	10315	23.8	31989	18.5	

Categorical variables given as number (percentage); continuous variable as mean ± standard deviation (SD).

*P values were acquired by Pearson’s chi-squared test if the variable was categorical, and by Student's t-test if the variable was continuous.

**Fig 2:**
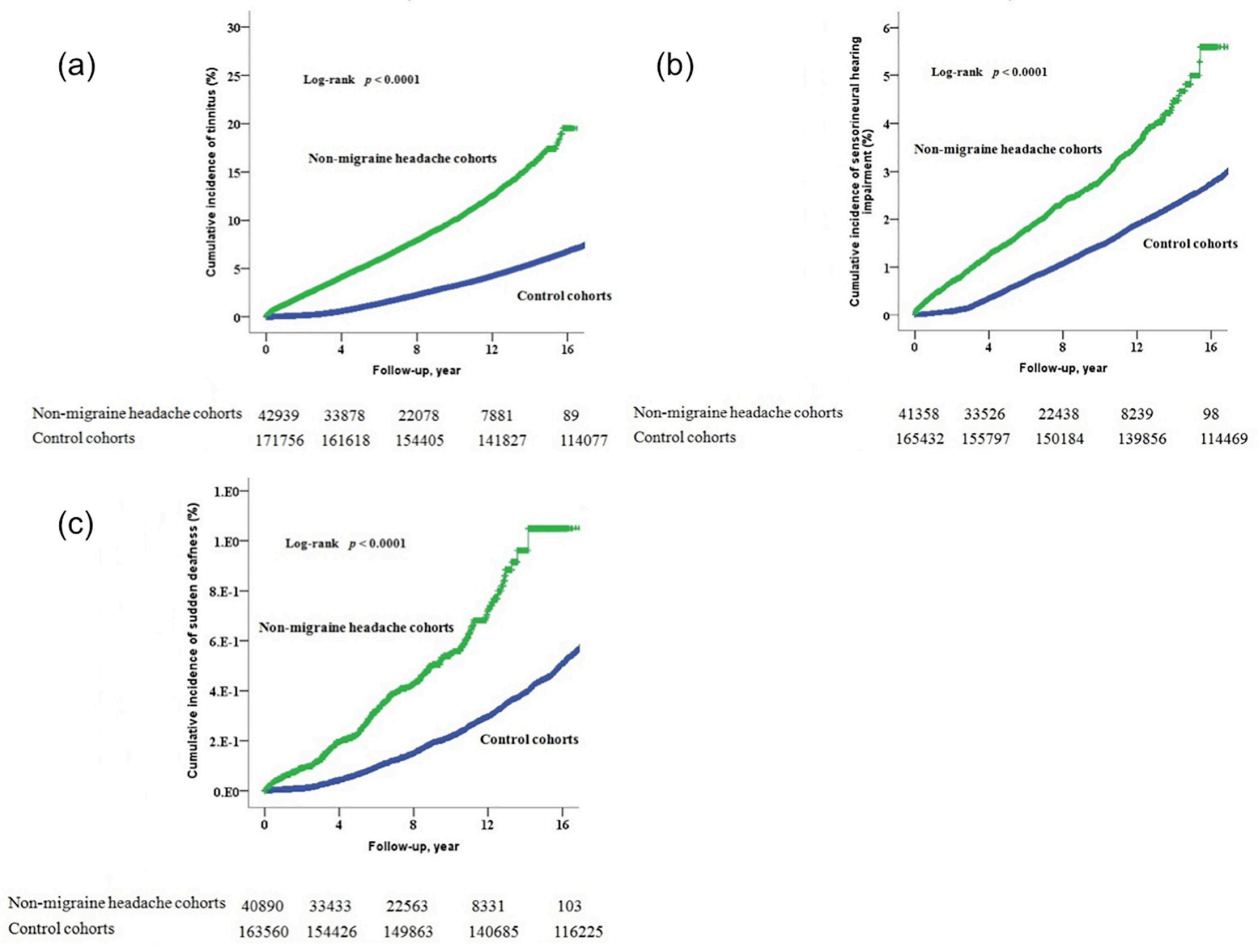
The cumulative incidence of individual tinnitus, sensorineural hearing impairment, and sudden deafness. The cumulative incidence of tinnitus (a), sensorineural hearing impairment (b), and sudden deafness (c) in the non-migraine headache cohort was significantly higher than that in the control cohort (log rank, p<0.001).

The mean duration of follow-up was 7.79 years for the non-migraine headache cohort and 14.49 years for the control cohort (p < 0.001). By the end of follow-up, the percentage of patients who also had tinnitus, sensorineural hearing impairment, or sudden deafness was 9.4% (95% CI, 2.96–3.21) in the non-migraine headache cohort and 7.7% (95% CI, 2.62–2.83) in the control cohort (p < 0.0001). The incidence of tinnitus, sensorineural hearing impairment, or sudden deafness was 121.1 per 10^6^ person-years of follow-up in the non-migraine headache cohort and 52.9 in the control cohort (p < 0.001). The cumulative incidence of each individual disorder (tinnitus, sensorineural hearing impairment, and sudden deafness) was significantly higher in the non-migraine headache cohort than in the control cohort (p < 0.001) (Fig 2).

The crude and adjusted hazard ratios (aHRs) for all 3 disorders combined (tinnitus, sensorineural hearing impairment, and sudden deafness) were 2.85 (95% CI, 2.75–2.96; p < 0.001) and 2.73 (95% CI, 2.62–2.84; p < 0.001), respectively (Table 2). As compared to the control cohort, the crude and adjusted hazard ratios (HRs) for the 3 disorders combined in the non-migraine headache cohort were 2.85 (95% CI, 2.75–2.96; p < 0.001) and 2.73 (95% CI, 2.62–2.84; p < 0.001), respectively. Age, sleep disorders, heart disease, and hypertension were also significantly associated with increased aHRs for all 3 disorders combined.

**Table 2:** Crude and adjusted hazard ratios for combining tinnitus, sensorineural hearing impairment, and sudden deafness.

Variable	Crude			Adjusted*		
	HR	95% CI	*p*-value	HR	95% CI	*p*-value
Non-migraine headache (yes/no)	2.85	2.75–2.96	<0.001	2.73	2.62–2.84	<0.0001
Sex (men/women)	1.69	1.63–1.74	<0.0001	0.97	0.93–1.01	0.10
Age (per year)	1.03	1.03–1.03	<0.0001	1.03	1.03–1.03	<0.0001
Comorbidities (yes/no)						
Sleep disorders	3.56	3.22–3.94	<0.0001	1.20	1.08–1.33	0.001
Heart diseases	3.95	3.65–4.28	<0.0001	1.31	1.19–1.44	<0.0001
Hypertension	3.83	3.52–4.16	<0.0001	0.82	0.74–0.91	<0.0001
Diabetes	4.01	3.55–4.53	<0.0001	1.04	0.91–1.19	0.55
Hyperlipidemia	4.00	3.62–4.41	<0.0001	1.10	0.98–1.23	0.10
Chronic kidney disease	4.34	3.15–5.96	<0.0001	1.20	0.87–1.66	0.26
Chronic hepatitis	3.46	3.11–3.85	<0.0001	1.12	1.00–1.25	0.06
Geographic region						
Northern	1	Reference		1	Reference	
Central	1.16	0.11–1.20	<0.0001	1.04	1.00–1.08	0.08
Eastern	0.98	0.88–1.09	0.74	0.91	0.82–1.02	0.09
Southern	1.09	1.05–1.13	<0.0001	0.96	0.93–1.00	0.05
Urbanization level						
Urban	1	Reference		1	Reference	
Suburban	1.06	1.02–1.10	0.001	1.00	0.97–1.04	0.94
Rural	1.15	1.10–1.19	<0.0001	0.98	0.93–1.02	0.33

Abbreviations: HR, hazard ratio; CI, confidence interval.

*Adjusted for all covariates (age per year, sex, comorbidities, geographic region, and urbanization level).

The crude HR and aHRs for each individual disorder as compared to the non-migraine cohort were 3.05 (95% CI, 2.91–3.19; p < 0.0001) for tinnitus, 1.89 (95% CI, 1.74–2.05; p < 0.0001) for sensorineural hearing impairment, and 2.14 (95% CI, 1.77–2.59; p < 0.0001) for sudden deafness in non-migraine headache cohort, respectively (Table 3).

**Table 3:** Crude and adjusted hazard ratios for individual tinnitus, sensorineural hearing impairment, and sudden deafness.

Variable	Crude			Adjusted*		
	HR	95% CI	*p*-value	HR	95% CI	*p*-value
For tinnitus Non-migraine headache (yes/no)	3.22	3.01–3.36	<0.0001	3.05	2.91–3.19	<0.0001
For SNHL Non-migraine headache (yes/no)	2.07	1.92–2.22	<0.0001	1.89	1.74–2.05	<0.0001
For sudden deafness Non-migraine headache (yes/no)	2.59	2.19–3.06	<0.0001	2.14	1.77–2.59	<0.0001

Abbreviations: HR, hazard ratio; CI, confidence interval; SNHL, sensorineural hearing impairment.

*Adjusted for all covariates (age per year, sex, comorbidities, geographic region, and urbanization level).

## Discussion

In this large-scale cohort study based on data from the Taiwan NHIRD, we investigated the risks of tinnitus, sensorineural hearing impairment, and sudden deaf associated with non-migraine headache. We found that patients with non-migraine headache are at significantly greater risk of tinnitus, sensorineural hearing impairment, and sudden deafness than are those without chronic headache.

TTH and migraine are the most common types of headache among the general population [1,4]. Although TTH and migraine are different clinical entities and demonstrate different somatosensory cortex excitability on magnetoencephalography [16], both TTH and migraine patients may experience sleep disturbances [17,18], neuroticism, and depression [19]. In addition, the laterality and severity of primary headache and tinnitus are significantly related [6]. Several cohort studies have shown a causal relationship between headache type and neurodegenerative diseases. For example, two cohort studies suggest that migraine increases the risk of tinnitus and/or sudden deafness [8,9]. Patients with TTH were found to be at increased risk for Parkinson's disease [20]. Here, we show that non-migraine headache is associated with higher risks of tinnitus, sensorineural hearing impairment, and sudden deafness.

This study excluded migraine and some types of secondary headache in the non-migraine headache cohort and excluded all types of headache in the control cohort. Thus, the non-migraine headache cohort mainly includes patients with TTH, medication overuse headache, temporal or giant cell arteritis, or idiopathic low cerebrospinal fluid pressure [21]. In addition, neck pain is highly prevalent in individuals with primary headaches (85.7%) and is also experienced by patients with idiopathic low cerebrospinal fluid pressure [21,22]. Previous studies have shown that specific subtypes of tinnitus (somatic tinnitus and cervicogenic somatic tinnitus) are associated with increased cervical and pericranial muscle tenderness in patients with TTH, migraine, myogenous pain, and arthrogenous temporomandibular joint disorders [7,10,11]. The observed increased risk of tinnitus, sensorineural hearing impairment, and sudden deafness in patients with history of non-migraine headache in our present study is consistent with these findings.

Poor sleep, hypoxia, neural inflammation, and oxidative stress damage are important factors in the pathogenesis of primary headache [17,18], tinnitus [23–26], sensorineural hearing impairment [27–30], and sudden deafness [31]. In addition, increased activation of the sympathetic nervous system in the limbic and autonomous brain regions [32] and central sensitization and/or cortical hyperexcitability have been observed in patients with chronic headache and tinnitus [33,34]. Thus, the mechanisms underlying non-migraine headache may be held in common with those of tinnitus, sensorineural hearing impairment, and sudden deafness.

This study has several limitations. Misclassification bias introduced by the use of ICD-9-CM might be present. Second, ‘migraine’ might be recorded as ‘headache’ in the dataset, increasing the rate of false-positive diagnoses in the non-migraine headache group. Thus, our conclusions should be interpreted conservatively. Third, unmeasured variables such as noise exposure or medication use may confound the study results.

## Conclusions

Taken together with the findings of previous studies, our results suggest that non-migraine headache is associated with increased risks of tinnitus, sensorineural hearing impairment, and sudden deafness. Therefore, clinicians should pay close attention to the history of headache when caring for patients with tinnitus and hearing impairment.
